# The climate crisis and healthcare: What do infection prevention and stewardship professionals need to know?

**DOI:** 10.1017/ash.2023.170

**Published:** 2023-08-02

**Authors:** Alison G. Freifeld, Alexandra I. Todd, Ali S. Khan

**Affiliations:** 1 Department of Medicine, University of Nebraska Medical Center, Omaha, Nebraska; 2 University of Nebraska Medical Center, College of Public Health, Omaha, Nebraska

## Abstract

The climate crisis calls for urgent action from every level of the US healthcare sector, starting with an acknowledgment of our own outsized contribution to greenhouse gas emissions (at least 8.5% of carbon emissions). As the climate continues to become warmer and wetter, the medical establishment must deal with increasing rates of pulmonary and cardiovascular diseases, heat-related illness, and emerging infectious diseases among many other health harms. Additionally, extreme weather events are causing healthcare delivery breakdown due to physical infrastructure damage, slowed supply chains, and workforce burden. Pathways for healthcare systems to meet these challenges are emerging. They entail significant measures to mitigate our carbon footprint, embrace shared and equity-driven governance, develop new metrics of accountability, and build more resilience into our care delivery processes. We call upon SHEA to play a unique leadership role in the fight for sustainable, equitable, and efficient health care in a rapidly changing climate that immediately threatens human well-being.

The climate crisis has unleashed a host of threats to human health worldwide. Burning energy-dense fossil fuels (eg, coal, oil, and gas) have allowed for unprecedented human achievements and powered the modern economy, but at the same time it has promoted the 2 main drivers of climate change: air pollution and atmospheric accumulations of carbon dioxide and other greenhouse gases (GHG). Dramatic increases in land and ocean mean surface temperatures, rising concomitantly with carbon emissions over the past 150 years, have led to novel weather patterns characterized by intense storms and flooding, extreme heat waves, and unpredictable cycles of prolonged drought and torrential rainfall. The undeniable evidence for these effects is laid out in the most recent report from the Sixth Intergovernmental Panel on Climate Change (IPCC6), published in the fall of 2021, which represents a consensus of 270 authors from 67 countries.^1^ Significant morbidity and mortality are directly and indirectly associated with climate-driven weather, especially extreme temperatures, tropical and coastal storms, and wildfires.^2,3^ A recent WHO report further indicated that well over 95% of people on Earth are exposed to poor air quality, and air pollution is now counted among the greatest causes of mortality worldwide.^4^ With ongoing high levels of fossil fuel combustion, cascading harms to environmental and human health are happening right now and will worsen in the near future. Furthermore, the damages are neither equally nor equitably distributed; they disproportionately affect the most vulnerable and least resilient populations among us: children, the elderly, ethnic minorities, poorer communities, and those with underlying health problems.^2–6^ Accordingly, the WHO declared in 2021 that climate change represents “the single biggest health threat facing humanity.”^4^ The calamity cannot be overstated. The IPCC6 has identified a 1.5°C rise in global temperatures over preindustrial levels as a tipping point at which widespread degradation of human and natural environments will be “impossible to reverse.”^1^ Having already exceeded 1.2°C of warming, a new UN Environment Programme report indicates that there is now “no credible pathway” to keep the rise in global temperatures below the key threshold of 1.5°C.^7^ Based on current levels of fossil fuel use, we are on track to exceed 2.5°C by the end of this century. Widespread harm to individual and public health, magnified by the rapid pace of climate change, is expected to accelerate, with the potential to overwhelm healthcare services worldwide (Figure 1).

**Figure 1.**
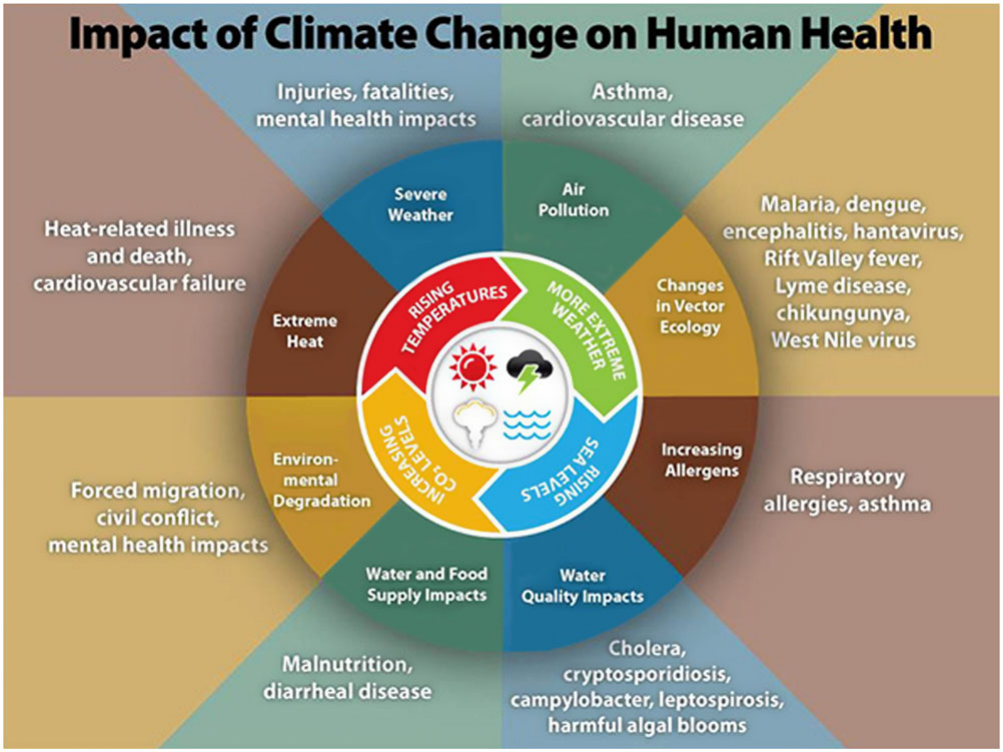
*Impact of Climate Change on Health*. A list of potential human health impacts from climate change. From the National Center for Environmental Health, Centers for Disease Control and Prevention, n.d. (https://www.cdc.gov/climateandhealth/effects/default.html).

The climate crisis calls for urgent action from every level of the US healthcare sector. First, we must acknowledge the impact of our own carbon footprint: the healthcare sector is among the most carbon-intensive services in the world and contributes significantly to global greenhouse gas emissions.^8,9^ Efforts to reduce that contribution are underway, prompted by proactive institutions across the country, but they need to expand dramatically and quickly. At the same time, we are challenged to understand and adapt to the climate-induced health threats to individuals and populations, while maintaining high-quality care delivery, even as the climate crisis rapidly unfolds. Herein, we propose that preparing for a harsh and unpredictable climate future starts by reimagining a healthcare system founded on foresight, planning, and resilience.

## Healthcare’s big carbon footprint

In 2014, an in-depth global study examined all Organization for Economic Cooperation and Development (OECD) countries (minus Chile), plus India and China, was the first to show that the healthcare sectors from the 36 countries combined were responsible for 1.6 gigatonnes of carbon dioxide (CO_2_) equivalent (GtCO_2_e) emissions or 4.4% of the total emissions from these nations.^9^ The countries studied represented 54% of the world’s population and 78% of world GDP, demonstrating the extensive impact of healthcare activities on global emissions. Put simply, if the healthcare industry was a country, it would be the fifth-largest greenhouse gas emitter on the planet.

A second, more comprehensive study of global health-sector emissions, also using 2014 data, was conducted by Healthcare without Harm (HWH), which is an international NGO working to transform the health sector worldwide into an ecologically sustainable enterprise.^10^ The HWH study replicated the original finding that health care accounts for 4.4% of global emissions. It went on to show that over half of that footprint comes from energy use. This includes direct greenhouse gas emissions associated with healthcare facility fuel combustion in boilers, furnaces, institutional vehicles as well as emissions due to the purchase of electricity, steam, heat, or cooling for system operations. The majority of energy use, however, is indirectly attributable to the production and transport of goods and services in health-care-sector supply chains. Not surprisingly, the US health sector was the world’s number one emitter in both absolute and per capita terms, accounting for 7.6% of our national footprint. That was in 2014. A recent update found that US healthcare-industry greenhouse gas emissions overall rose 6% from 2010 to 2018, now contributing ∼8.5% of domestic greenhouse gas emissions.^8^ Importantly, this translated into a loss of 133,000–188,000 disability-adjusted life years (DALYs) in 2018. Ironically, while the healthcare sector is positioned on the front lines of the climate crisis, coping with an array of climate-related illnesses, it is also a significant contributer to the actual cause of that crisis.

## Emerging infections driven by climate change

The wide range of health harms posed by climate change vary by geography, emissions sources, population vulnerability, and regional or national adaptability and resilience. Air pollution is by far the most significant and widespread climate threat across the globe, with >7 million people a year dying from exposure to outdoor air pollution primarily caused by burning fossil fuels: 1 in 8 of all deaths globally.^4^ In the United States, exteme heat is the deadliest weather-related hazard. Heat-related mortality rates have been rising in recent years with escalating frequency and duration of brutal heatwaves stemming from the warming effects of 200 years worth of accumulated greenhouse gases.^1–3^ Infectious diseases due to emerging and re-emerging pathogens are increasingly recognized as an important indirect consequence of anthropogenic climate change.^11,12^ These can be categorized as (1) “spillover infections,” primarily viruses that are derived directly from animal or, most often, bat sources; (2) vectorborne infections transmitted by arthropod vectors such as ticks and mosquitoes; and (3) bacterial infections (as well as other infections) emerging as a consequence of changing climatic conditions.

### “Spillover” viruses

Spillover infections, primarily due to viruses, are those that occur naturally in animals but, due to population movements of both animals and humans, have “jumped species” and have become capable of causing human infections.^12^ Waves of human outbreaks due to viruses that were originally established in wild or domestic animal reservoirs have been observed in the early 21st century: Middle East respiratory syndrome (MERS), severe acute respiratory syndrome (SARS), the 2009 pandemic influenza H1N1, and the COVID-19 pandemic are prominent examples.

Climate change and rapid human population growth are the intertwined problems that drive this spillover trend. Both promote intensified contact between human beings and animal reservoirs, enhancing opportunities for pathogens typical in animal and insect vectors to become invasive to human populations.^12–14^ As the Earth warms, especially in equatorial regions, animal and insect vector species seek cooler habitats in more temperate zones north of the equator or at higher altitudes. At the same time, people are moving toward these same regions, seeking refuge from rising sea levels and punishing cycles of heat, drought, and flooding. Demands for increased food production intensify with the growing human population, leading to damaging land-use policies that are linked to emerging zoonotic diseases.^15,16^ Deforestation to clear land for agricultural and ranching purposes destroys natural habitats, forcing wildlife (and their pathogens) into more populated areas. The sum of these human activities is an expansion of opportunities for spillover viruses from animal sources.^11–18^

Modeling by Carlson et al^18^ has indicated that “This ecological transition may already be underway, and holding warming under 2°C within the 21st century will not reduce future viral sharing.” Bats, they added, account for the majority of novel viral sharing due to their widespread global distribution and their ability to host a variety of viruses.

Ebola virus disease is perhaps the most notorious example of a spillover virus. Close associations between the wild animals that host Ebola, primarily nonhuman primates and bats, and people who hunt and eat them (ie, bushmeat), or encroach on their habitats by forest destruction and urbanization, allow for the jump from animals to humans. Periodic human outbreaks in endemic regions of sub-Saharan Africa as well as in nonendemic areas of the globe—have heightened public health conerns about broader Ebola outbreaks in human populations as spillover conditions accelerate.

### Arthropod-borne infections

Arthropod-borne infections are increasing worldwide, and this expansion is attributed to a combination of increased urbanization, global travel, and environmental changes. Specifically, warming temperatures and increased precipitation trends that favor vector reproduction, feeding, and location play a large role.^18–20^ *Aedes aegypti*, the principal mosquito vector of many arboviral diseases including dengue, chikungunya, yellow fever, and Zika, has spread globally in recent decades, potentially exposing almost half of the world’s population to these diseases. Dengue virus has extended it range well beyond its traditional tropical and subtropical geography in the 21st century, with transmission and outbreaks seen in parts of the United States, southern Europe, South America, and Australia.^18^ Chikungunya (2013) and zika (2015) infections also surprisingly emerged in temperate regions in the Americas. Warming temperatures, as well as both excessive rainfall and drought conditions, play a critical role in the optimal development, lifespan, and disease transmission capabilities of *Aedes* mosquitoes. Comparably, robust data suggest that long-term range expansion has been occurring for the *Anopheles* mosquito vectors of malaria in Africa over the last century.^20^ The establishment of mosquito vectors at higher elevations (documented in both Africa and South America) and into more temperate zones, farther from the equator, will likely drive the expansion of malarial disease in those regions. Awareness of these geoclimatic alterations is essential in diagnosing arthropod-borne infections in patients coming from regions not traditionally considered endemic.

In the United States, West Nile virus fever (WNV) and Lyme disease (*Borrelia burgdorferi*) are the 2 most prevalent vectorborne diseases. Both were initially reported in the northesastern United States in the late 20th century, their epidemiological “march” westward. This pathogenic success is owed to warmer and wetter weather patterns that promote the survival and range expansion of their vectors, *Ixodes* spp ticks and *Culex* spp mosquitoes.^21,22^ West Nile Virus has an especially significant endemic presence in the United States since its introduction from Europe to New York in 1999. Sporadic cases as well as larger outbreaks occur each summer, scattered geographically across the country, depending on local conditions that support *Culex* mosquito populations. In the summer and fall of 2021, the largest identified single West Nile outbreak in US history (>1,600 cases) occurred in southwestern Arizona, linked to wetter-than-average rainfall.^23^

Rising incidences of geographically itinerant arthropod-borne infections will place added burdens on medical systems. Most of these infections present as unexplained febrile illnesses and may prompt empirical antimicrobial use. Rapid and accurate diagnostic test results from multiplex polymerase chaing reaction (PCR) platforms could reduce the need for starting empiric antibiotics or antivirals but are typically costly. However, the high expense and uneven availability of such tests, especially in smaller and more rural hospitals, are likely to complicate such stewardship pressures.

### Bacterial (and other) infections and antimicrobial resistance

The connection between emerging and re-emerging antibiotic-resistant bacterial infections and climate change is less well defined, although there are supportive lines of evidence.^12,13^ Antibiotic resistance in bacteria develops largely under the selective pressure of antibiotic use and is then spread person to person. Other factors, however, may contribute to population-level increases in antibiotic resistance including, for example, the overuse of antibiotics in livestock farming. In a 2018 study published in *Nature Climate Science*, McFadden et al^24^ explored the role of climate (temperature, primarily) and other factors on the distribution of antibiotic resistance in human infections across the United States. In a data set of 1.6 million infections due to *Escherichia coli, Klebsiella pneumoniae*, and *Staphylococcus aureus*, they identified significant associations between antimicrobial resistance patterns in these common pathogens and higher local minimum temperatures.^24^ Another recent study similarly showed modest but significant impacts of both local temperature and humidity on surgical-site infection (SSI) rates within a representative data set in the continental United States, 2007–2014.^25^

Locoregional maximum daily precipitation and humidity, and minimum daily temperature were all significant predictors of SSI diagnosed after discharge. Climate modeling in this report further showed that small but meaningful increases in postdischarge SSIs can be anticipated over the next several decades if overall carbon emissions are not significantly reduced, with the Southeast region being particularly vulnerable. The broad epidemiologic associations in these reports do not imply causality, of course. However, they suggest that greenhouse-gas driven increases in ambient temperatures (and humidity in the study of SSIs) probably contribute to the rising prevalence of common hospital pathogens in the United States, including antibiotic-resistant strains. The implications are notable for infection control practitioners.

Exposure to air pollution poses a well-documented increased risk for the development of tuberculosis in endemic regions.^26^ Increased mortality in newly treated TB patients has also been linked to breathing higher concentrations of air pollution components (particulate matter, in small PM_2.5_ and large PM_10_ sizes, and noxious gases NO_2_, SO_2_, and O_3_) in a recent Chinese study.^27^ Wildfires represent a significant cause of exposure to ambient air pollution in the United States and are increasingly frequent due to climate change. Wildfire smoke is laden with particulate-matter lung irritatants, PM_2.5_ and PM_10_, that may exacerbate pulmonary diseases, including tuberculosis. This concern was affirmed in a recent study of patients with active tuberculosis disease from 8 California counties between 2014 and 2019 (n = 6,238 participants aged ≥15 years) in which wildfire-associated events, as determined by particulate matter monitoring across the regions, were associated with 23% higher odds of tuberculosis diagnosis over a 6-month observation period.^28^ This finding should prompt practitioners to consider wildfire air pollution as a risk for active tuberculosis disease in the United States.

Cholera disease is predictably magnified by warmer and wetter climatic conditions that favor *Vibrio cholerae* growth. The WHO recently reported that a global surge in cholera outbreaks over the past was “unprecedented,” with more frequent, larger, and more deadly than in years past. They appear related to major floods, unprecedented monsoons and cyclones promoted by climate change, combined with the known risk factors of poverty, clean water scarcity, and inadequate sewage handling and water treatment.^29^ Other waterborne diseases (both bacterial and nonbacterial) including leptospirosis, hepatitis A, cryptosporidium, norovirus and other diarrheal pathogens, are anticipated become more prevalent worldwide as a consequence of climate-change hazard risks combined with sociodemographic trends of urbanization, poverty, overcrowding and mass human displacement due to natural disasters and conflict.

Nonbacterial infections such as primary amebic encephalitis (*N. fowleri*), and the fungi *Candida auris* and *Cryptococcus gattii,* have emerged or re-emerged in recent years, and some evidence links them to altered climate patterns.^20^ These unusual pathogens are typically more difficult to diagnose and may be intrinsically resistant to common antimicrobials.

## Impacts of climate change on health systems

Healthcare-built environments are vulnerable to physical and infrastructure damage during extreme weather events, generally requiring quick action by hospital administration, including those in hospital epidemiology, to maintain safe and effective clinical operations. In 2005, Hurricane Katrina brought widespread flooding to New Orleans due to heavy and prolonged rains, coupled with a huge storm surge that breached the levees. Hospitals across the city were swamped by several feet of water. At Tulane University hospital, emergency generators ran out of fuel after 36 hours. “Temperatures in the hospitals soared into the upper 90s and conditions were made intolerable by 100% humidity and backed-up sewage. For several days, faculty, residents, nurses, and hospital personnel cared for patients in appalling conditions,” with staff hand-ventilating critical patients in shifts and evacuating patients to the nearby hospital garage.^30^ Bellevue Hospital, the oldest in New York City, experienced similar catastrophic breakdowns during Superstorm Sandy in 2012: extensive flooding of the building; sewage and trash backups; a prolonged power outage; and limited clean water with which to wash, drink, or perform life-sustaining procedures.^31^ These dramatic examples reflect the fragility of low-lying coastal hospitals where storm surges and high tides are tangible threats. However, devastating flooding can occur inland as well; in Nebraska in 2019, a severe winter storm triggered unprecedented flooding and billions of dollars in property damage. Notably, people who were injured or ill struggled to gain access to hospitals due to flooded or blocked roadways; 4 people died in that Midwestern catastrophe.^32^

Flooding of medical facilities and surrounding communities following heavy rains is associated with myriad infectious diseases during the weeks after such catastrophes, posing unique issues for infection control practitioners, as reviewed comprehensively by Paterson et al.^33^ Contaminated floodwaters within hospital spaces will increase risks for transmission of sewage-borne pathogens. Limitations in clean water for routine hand hygiene and cleaning medical instruments and surfaces could promote transmission of hospital-acquired infections, although there is no direct evidence of this happening routinely. Legionnaires disease a special concern. Although cases are typically sporadic, a recent study reported a 32% increase in monthly hospitalizations for Legionairre’s disease at US sites that experienced cyclonic storms, compared to sites in months without storms at sites that experienced “cyclonic” storms, compared to sites in months without storms.^34^ Mold infections following hospital flooding is an obvious threat, particularly for immunocompromised patients.^35^

## Healthcare preparedness (or lack of) during the COVID-19 pandemic

Much has been written, debated and regretted surrounding the issue of unpreparedness during the recent pandemic. Although a deadly pandemic had been predicted by scientists for decades, leaders in medicine and government were astonishingly ill-equipped to cope with the explosion of severe acute respiratory coronavirus virus 2 (SARS-CoV-2) infections globally. The medical sector failed to plan for workforce stresses and shortages, communication fiascoes, and supply-chain breaches that complicated care delivery.^36^

In the United States, concomitant climate-driven natural disasters further compromised healthcare access and delivery at the height of the pandemic: a deadly heat wave in the Pacific Northwest in 2020 and 2021; catastrophic floods in California and Louisiana; a deadly freeze event in Texas causing widespread days-long losses of power and water; and violent storms and tornados across the country all may have contributed to excess morbidity and mortality within COVID-19–stressed healthcare facilities.^37^ Thus, while the COVID-19 pandemic revealed an unexpected level of fragility within the American healthcare system, climate-related events compounded the issues of being unready. These cumulative failures should now swiftly translate into lessons for the healthcare sector, stimulating preparations to build resilience and redundancy into existing systems. Preparedness plans are necessary not only to meet the challenges of a next pandemic but also to address the immediate and near-future health impacts incurred by the current climate crisis.

## Hospitals (including infection control practitioners) must respond to climate change

A “perfect storm” created by the intersecting effects of climatic change, infectious pathogen threats, and inadequacies in current healthcare infrastructure is now on our horizon. How adeptly we address and correct obvious flaws in existing healthcare frameworks will have important consequences for the future of human health and well-being. We propose that the connections between climate change, novel infectious diseases, and healthcare are important to an understanding of how health systems in the United States can become more adapted and resilient as the climate crisis advances virtually unchecked and how we can mitigate the damage. Our collective challenge is to create a climate-resilient health system: one that can anticipate, respond to, cope with, recover from, and adapt to climate-related shocks and stress and, ultimately, bring sustained improvements in population health despite an unstable climate.^38^ In this context, SHEA is uniquely positioned to actively participate in partnerships that are developing pathways to healthcare resiliency. SHEA’s history and impeccable reputation for advancing the science of healthcare epidemiology—and translating that knowledge into effective policy and practice—would bring invaluable strengths to the all-hands-on-deck approach needed to address climate change (Box 1).

**Box 1.** Infection Control Contributions to a Resilient Healthcare Systems

Infection Control and Stewardship Professionals Roles	Actions and Goals
Health systems assessment	• Review potential infections that may emerge in the healthcare system as a consequence of climate events • Include representation from all sectors of the healthcare system
Preparation planning for public health emergencies	• Develop plans to maintain basic levels of essential healthcare delivery during rapidly evolving circumstances and resource scarcity. o Altered standards of care for infection control practices, eg, personal protective equipment shortages o Plan strategies to deal with critical antimicrobial medication and vaccine shortages
Partnerships: Collaboration and coordination	• Establish and strengthen partnerships within and outside of the system in preparation planning, ie, local health departments, first responders, etc • Develop coordinated plans for various disaster or public health scenarios • Provide clear, calm, consistent messages to staff and public during infection crises

Adapted from: Nuzzo JB, Meyer D, Snyder M, et al. What makes health systems resilient against infectious disease outbreaks and natural hazards? Results from a scoping review. *BMC Public Health* 2019;19:1310.

Preparedness for local climate-driven events is reflected in current Centers for Medicare & Medicaid Services (CMS) regulations requiring that facilities adopt emergency management programs and plans. The CMS identified 3 key essentials maintaining access to healthcare during disasters or emergencies: safeguarding human resources, maintaining business continuity, and protecting physical resources. CMS expanded their concerns in the recent Fiscal Year 2023 Inpatient Prospective Payment System proposed rule (August 2022), in which feedback was sought on how to maintain uninterrupted patient services due to climate threats and catastrophic weather events, with particular attention to underserved populations that are disproportionately harmed by climate change. Additionally, they urged the broader healthcare sector to evaluate and reduce its outsized contribution to greenhouse gas emissions.

Abundant information is available to guide assessments and planning for resilience in healthcare systems (Box 2). Practitioners who work in hospital epidemiology roles can play an integral part in this process by helping to identify threats and vulnerabilities to the system and by collaboratively preparing for rapid, nimble responses on an institutional basis. There are some specific ways hospital epidemiologists can be of great help. First, they can lend valuable expertise in developing institution-wide guidance for preventing and containing threats and in communicating that guidance to staff in a straightforward, evidence-based manner. Second, hospital epidemiologists should continue to strengthen ties with community health practitioners and health departments and become integrated into regional public health planning for adverse weather and climate events. During the COVID-19 pandemic, such liaisons between hospital epidemiologists and local health departments emerged quickly to coordinate community resources and responses. Ongoing collaborations with community surveillance activities could provide early warnings about impending impacts on the local population that might affect hospital resources, including increased emergency room visits, hospital bed utilization, and staff requirements on a surge basis. Third, a re-evaluation of current infection control interventions should be undertaken, with the goal of reducing the carbon footprint at each healthcare facility. For example, personal protective equipment is both costly to purchase and carbon-intensive to manufacture, but it is used broadly, sometimes for even minimal patient interactions. The infection control community could reduce PPE us by re-evaluating the science base on which transmissible diseases actually require contact isolation precautions and which do not. For example, as transmission of COVID-19 is now established, it would be important to explore whether or not isolation gowns are still required to reduce nosocomial spread and protect healthcare staff. Reassessing optimal use cultures and rapid tests for infection control purposes might also lead to decreased use of materials. Opportunities to reduce energy use and waste in the healthcare setting will become increasingly clear as more institutions engage in the HHS Healthcare Sector Climate Pledge (https://www.hhs.gov/climate-change-health-equity-environmental-justice/climate-change-health-equity/actions/health-care-sector-pledge/index.html) which commits them to take an inventory of their carbon footprint and begin active mitigation. A leader in this effort is the Practice Green Health organization (https://practicegreenhealth.org/), which will guide member institutions through the carbon emissions accounting and decarbonization processes.

Box 2. Tool Kits for Creating Climate Resiliency in Healthcare Systems

- US Department of Health and Human Services’ *Sustainable and Climate Resilient Health Care Facilities Initiative* (SCRHCFI) https://toolkit.climate.gov/topics/human-health/building-climate-resilience-health-sector
- World Health Organization (WHO) https://www.who.int/teams/environment-climate-change-and-health/climate-change-and-health/capacity-building/toolkit-on-climate-change-and-heal
- Pan American Health Organization’s *Smart Hospitals Toolkit*
- Canada’s *Health Care Facility Climate Change Resiliency Toolkit*
- World Bank Group’s *Climate Change and Health Diagnostic*

## Opportunity for a unique partnership

Creating climate-resilient health systems is critical, complex, and daunting because climate change is a problem with no clear boundaries. Its harmful effects are global, amplifying and stretching far into the future. Traditional healthcare emergency management practices cannot encompass the entirety of the threats it poses to human health and healthcare. Accordingly, a transformational process is required, one that engages healthcare practitioners and partners at every level and sector of the enterprise and is patient-centered and guided by equity and justice values. Institutional governance and leadership within healthcare systems must think and act quickly and creatively to make necessary changes. The scope of the planning, assessments, research, and programmatic activities to meet this challenge, coupled with the climate-driven emergence of infectious diseases, aligns well with the experience and expertise of those knowledgeable in healthcare epidemiology. The SHEA would be a natural association to host the comprehensive needs to create climate-resilient health systems.
